# METTL3-m6A methylation inhibits the proliferation and viability of type II alveolar epithelial cells in acute lung injury by enhancing the stability and translation efficiency of Pten mRNA

**DOI:** 10.1186/s12931-024-02894-z

**Published:** 2024-07-15

**Authors:** Qiuyun Wang, Jie Shen, Shiyuan Luo, Zhize Yuan, Shiyou Wei, Qiang Li, Qianzi Yang, Yan Luo, Lei Zhuang

**Affiliations:** 1 Department of Anesthesiology, Ruijin Hospital, Shanghai Jiaotong University School of Medicine, Shanghai, China; 2 Department of Thoracic Surgery, Shanghai Pulmonary Hospital, Tongji University School of Medicine, Shanghai, 200433 China; 3 Department of Anesthesiology, Shanghai Pulmonary Hospital, School of Medicine, Tongji University, Shanghai, 200433 China; 4 Outcomes Research Consortium, Cleveland, OH USA

**Keywords:** Acute lung injury, Methyltransferase-like 3, N6-methylation of adenosine, Phosphatase and tensin homolog

## Abstract

**Background:**

The pathogenesis of acute lung injury (ALI) involves a severe inflammatory response, leading to significant morbidity and mortality. N6-methylation of adenosine (m6A), an abundant mRNA nucleotide modification, plays a crucial role in regulating mRNA metabolism and function. However, the precise impact of m6A modifications on the progression of ALI remains elusive.

**Methods:**

ALI models were induced by either intraperitoneal injection of lipopolysaccharide (LPS) into C57BL/6 mice or the LPS-treated alveolar type II epithelial cells (AECII) in vitro. The viability and proliferation of AECII were assessed using CCK-8 and EdU assays. The whole-body plethysmography was used to record the general respiratory functions. M6A RNA methylation level of AECII after LPS insults was detected, and then the “writer” of m6A modifications was screened. Afterwards, we successfully identified the targets that underwent m6A methylation mediated by METTL3, a methyltransferase-like enzyme. Last, we evaluated the regulatory role of METTL3-medited m6A methylation at phosphatase and tensin homolog (*Pten*) in ALI, by assessing the proliferation, viability and inflammation of AECII.

**Results:**

LPS induced marked damages in respiratory functions and cellular injuries of AECII. The m6A modification level in mRNA and the expression of METTL3, an m6A methyltransferase, exhibited a notable rise in both lung tissues of ALI mice and cultured AECII cells subjected to LPS treatment. METTL3 knockdown or inhibition improved the viability and proliferation of LPS-treated AECII, and also reduced the m6A modification level. In addition, the stability and translation of *Pten* mRNA were enhanced by METTL3-mediated m6A modification, and over-expression of PTEN reversed the protective effect of METTL3 knockdown in the LPS-treated AECII.

**Conclusions:**

The progression of ALI can be attributed to the elevated levels of METTL3 in AECII, as it promotes the stability and translation of *Pten* mRNA through m6A modification. This suggests that targeting METTL3 could offer a novel approach for treating ALI.

**Supplementary Information:**

The online version contains supplementary material available at 10.1186/s12931-024-02894-z.

## Introduction

The pathophysiology of acute lung injury (ALI) and acute respiratory distress syndrome (ARDS) involves disruption of epithelial barrier, causing an influx of fluid rich in proteins and the accumulation of neutrophils within the alveolar space, which ultimately leads to respiratory distress and a decrease in oxygen levels [1]. This results in increased mortality rates and elevated healthcare costs [2]. release inflammatory substances to contribute to the development of systemic inflammatory response syndrome and coagulation imbalance in ALI [3]. After ALI, the repair process involves the proliferation of AECII cells and their transformation into AECI cells as well as interstitial cells. These cellular changes play a crucial role in facilitating lung injury recovery and reducing fibrosis [4]. Therefore, enhancing the proliferation and activation of AECII is regarded as a viable approach for treating ALI.

N6-methylation of adenosine (m6A) is a commonly occurring mRNA modifications in eukaryotes that impacts various aspects of RNA metabolism, such as splicing, decay, export, and translation [5]. M6A methylation isregulated by a combination of methyltransferase complexes, including methyltransferase-like 3 (METTL3), methyltransferase-like 14, (METTL14), WT1-associated protein (WTAP). Additionally, demethylases such as fat mass and obesity-associated protein (FTO) and alkB homolog 5 (ALKBH5) also play a role in the co-regulation process. [6]. The m6A modification holds significant significance in the regulation of gene expression and preservation of genome stability, playing a crucial role in diverse pathological conditions, including brain disorders, cancers, excessive weight gain, and coronary artery disease [7–10]. Previous studies reported the involvement of m6A in ALI [11, 12], but the exact mechanism is still unclear.

Phosphatase and tensin homolog (PTEN) serves as a crucial tumor suppressor, playing a pivotal role in regulating cell viability and proliferation by antagonizing the phosphoinositide-3 kinase (PI3K)/Akt pathway [13]. PTEN is essential for preserving the structural integrity of the airway epithelium, thereby playing a crucial role in the pathological progression of ALI [14]. In the current study, we intent to explore the impact of m6A on the viability, proliferation, and inflammation of ACEII during LPS-induced cellular injury. Additionally, we aimed to elucidate the underlying molecular mechanism through which METTL3 interacts with Pten mRNA to regulate the outcomes of ALI in vivo and exposure of AECII to LPS in vitro.

## Materials and methods

### Ethical statement

This study was conducted following the approved protocol by the Institutional Animal Care and Use Committee of Ruijin Hospital (Shanghai Jiao Tong University School of Medicine). All procedures related to animal handling strictly adhered to the guidelines outlined in the publication *Guide for the Care and Use of Laboratory Animals* issued by the National Institute of Health (Bethesda, MD, USA).

### Animal experiments

Male C57BL/6 mice, aged 4 to 6 weeks and weighing approximately 18 to 22 g, were utilized in this study. These mice were obtained from Shanghai Jiao Tong University (animal license number SCXK (hu) 2017-0011) and bred in animal facility under specific pathogen-free conditions. The animals had *ad libitum* access to food and water, while their bedding was regularly replaced. The holding room was kept within a controlled temperature range of 18–22 °C and maintained at a relative humidity level between 50 and 60%. The animals were categorized into two groups randomly and received intraperitoneal injections of either isovolumetric saline or 0.5 mg/ml LPS at a dose of 5 mg/kg. The animals were euthanized for tissue sampling 6–12 h after the injection following anesthesia induction with 1% pentobarbital.

### Measurement of wet-to-dry (W/D) ratio of the lungs

After washing the lung tissue with normal saline, cooled to 4 °C, drained off, and weighed. The lung tissue was subsequently subjected to a drying process at 60 °C for 48 h, followed by reweighing. The W/D was calculated by the ratio of two weights.

### AECII culture and LPS treatment

AECII were obtained from the lungs of C57BL/6 mice using a solution called Hanks Balance Salt Solution (HBBS; Sigma, St Louis, MO), c-Kit (Santa Cruz Biotechnology, Santa Cruz, CA), collagenase (1 mg/ml in HBSS; Sigma), 0.1% trypsin (Gibco, Carlsbad, CA), and coated beads (Thermo Fisher Scientific, Waltham, MA). AECII were identified by immunofluorescence assay, and AECII were CK18(CY5757, Abways, Shanghai, China) positive and Vimentin (BF8006, Affinity, Changzhou, China) negative. Briefly, cells were seeded in a 48-well plate at a concentration of 0.5 × 10^5^ cells/well. After 4% PFA fixation, 0.5% Triton-100 penetration and 3% BSA blockage, the cells were incubated overnight at 4 °C with primary antibodies against CK18 and Vimentin, respectively. Twenty-four hours later, the samples were subjected to secondary antibody incubation (Abways, Shanghai, China) followed by Hoechst staining and visualized under a fluorescence microscope. The AECII were cultured in DMEM/F12 medium (Gibco, MA, USA.) supplemented with 10% fetal bovine serum (FBS, HyClone, Utah, USA.). All culture media contained streptomycin at a concentration of 100 µg/ml and penicillin at a concentration of 100 U/ml. Subsequently, AECII were incubated with different concentrations of LPS (5, 10, 20–40 µg/mL) for a duration of 12 h in a cell culture incubator under an atmosphere consisting of 95% air and 5% CO2 maintained at a temperature of 37 °C. Cells of less than six generations were used in the experiments.

### Hematoxylin/eosin and Masson’s trichrome staining

The lungs were embedded in paraffin, sectioned into thin slices, and then stained with hematoxylin/eosin (Sangon Biotech Co. Ltd.) for 10 min and 2 min, respectively. For Masson’s trichrome staining, lung sections were incubated with the Masson collagen staining reagent and subsequently immersed in xylene for a duration of 5 min.

### Plasmid construction, lentivirus production, and cell transfection

Lentiviral vectors and short hairpin RNA (shRNA) were designed and synthesized by a commercial supplier. *Mettl3* (NCBI Reference Sequence: NM_019721.2) and *Pten* (NCBI Reference Sequence: NM_008960.2) cDNAs were cloned and inserted into the EcoRI and BamHI sites of the lentivirus plasmid pLVX-puro. *Mettl3* and *Pten* shRNAs were designed using RNAi Express (http://rnaidesigner.thermofisher.com/rnaiexpress/). The shRNAs were then inserted into the AgeI and EcoRI sites of the lentiviral plasmid pLKO.1. Lipofectamine-3000 (Invitrogen) was utilized for transfection of the plasmids and shRNAs into AECII following to the manufacturer’s protocols. pHIV plasmids with full gene sequence of *Pten* (Generay, Shanghai, China) and *Mettl3* (Generay, Shanghai, China) were inserted into the AgeI and EcoRI sites of the lentiviral plasmid pLKO.1, which were then transfected into AECII. The AECII were harvested within 72 h for subsequent analysis. The primer sequences utilized in this study are provided in Table 1.

**Table 1:** Primers used for RT-qRCR

Gene name		Sequence
Actin	Forward	5’- GGCTGTATTCCCCTCCATCG − 3’
	Reverse	5’- CCAGTTGGTAACAATGCCATGT − 3’
METTL3	Forward	5’- TCATCTTGGCTCTATCCGGC-3’
	Reverse	5’- CGTGTCCGACATCCTAGCTC-3’
METTL14	Forward	5’- GTGGTCGGGAAAGAAACCGA-3’
	Reverse	5’- AGCTCTGAAGCAAGTCTCCA-3’
WTAP	Forward	5’- AGGACATTTTGTGTAGGGTCAAGT-3’
	Reverse	5’- GTGAGGAAGAGTGCCCTGAC-3’
FTO	Forward	5’- GCTCACAGCCTCGGTTTAGT-3’
	Reverse	5’- GTCGCCATCGTCTGAGTCAT-3’
ALKBH5	Forward	5’- AGATTGGTCACTGACACCCC-3’
	Reverse	5’- GCAGCTTCTCTACCAAGCCA-3’
PTEN	Forward	5’- ATCTTGTGCTCACCCTGACA − 3’
	Reverse	5’- AGCCTCTGGATTTGATGGCTC − 3’
shMETTL3-1	Forward	5’- GCATCATCTCTAAACCTAAGA-3’
shMETTL3-2	Forward	5’- GCACATCCTACTCTTGTAACT-3’
shPTEN-1	Forward	5’- GCAGCTAGAGTGAGTATATTT-3’
shPTEN-2	Forward	5’- GCACGAATAATAAGGCATTGA-3’

### RNA isolation and quantitative real-time PCR

TRIzol reagent (Life Technologies, MA, USA) was utilized to extract the total RNA from the lung tissue and AECII following the manufacturer’s protocol. cDNA was produced by using a cDNA synthesis kit (Bio-Rad, Beijing, China). Quantitative real-time PCR was conducted on a 7500 Fast Real-Time PCR System (Applied Biosystems, MA, USA), using Powerup SYBR Green PCR Master Mix (Life Technologies, MA, USA). Data analysis was performed using the 2^−ΔΔCT^ method. The expression levels of genes were normalized by that of β-actin mRNA. The primer sequences utilized in this study can be found in Table 1.

### Western blotting

Total protein fractions of lung tissues and AECII were collected according to the manufacturer’s protocols (KeyGEN BioTech, Jiangsu, China). The total protein concentrations were determined using the BCA protein detection assay kit (Beyotime Biotechnology, Beijing, China). Subsequently, 30 µg of protein lysate was loaded onto a 10% polyacrylamide gel for electrophoresis. It was then transferred to polyvinylidene difluoride membranes and incubated with primary antibodies including anti-m6A (ABclonal, A17924), anti-METTL3 (Abcam, AB195352), anti-METTL14 (Cell Signaling, 51,104 S), anti-WTAP (Santa Cruz, Lot#B2578), anti-FTO (Cell Signaling, 45,980 S), anti-ALKBH5 (Abcam, AB195377), anti-PTEN (ABways, CY5862), anti-β-actin (ABways, AB0055), anti-eIF3A (Proteintech, 10291-1-AP), and anti-CK18 (ABways, CY5757). Finally, immunoreactivity was visualized using an electrochemiluminescence kit (Junxin Biotech, Suzhou, China).

### Cell counting kit-8 analysis

AECII were seeded into 96-well plates (Corning Inc., Corning, NY, USA) at a density of 2 × 10^5^ cells per well in triplicates for each experimental condition. On the following day, the cells were incubated with either vehicle or LPS (Sigma) at varying concentrations (5, 10, 20, or 40 µM) and cultured under an atmosphere of 95% air and 5% CO_2_ at a temperature of 37 °C for 24 h. Subsequently, each well was supplemented with 10 µL of the cell counting kit-8 (CCK-8; Vazyme Biotech, Nanjing, China) reagent and further incubated at a temperature of 37 °C for an additional 2 h. The absorbance at 450 nm was measured using a microplate reader (Beckman).

### EdU incorporation assay

The detection of cell proliferation was performed using 5-Ethynyl-2′-deoxyuridine (EdU, Junxin Biotech, Suzhou, China) as per the manufacturer’s protocols. Briefly, AECII were seeded at a density of 1.5 × 104 cells per well in 24-well plates. Following exposure to various treatments, each well was supplemented with EdU (10 µL) and incubated for 2 h. Subsequently, the cells were fixed with 4% paraformaldehyde at room temperature for 30 min and treated with 0.1% Triton-100 before staining with the Apollo staining reaction liquid. Nuclei were counterstained using Hoechst stain. The results of the EdU assay were visualized under a fluorescence microscope (Zeiss).

### Dual-luciferase reporter assay

The m6A modification of *Pten* mRNA was predicted by utilizing the m6A prediction tool available at http://www.cuilab.cn/sramp. To construct the *Pten* wild-type reporter (*Pten*-WT), full-length *Pten* cDNA was cloned into the psiCHECK-2 luciferase reporter plasmid (Promega, Madison, USA). In order to generate the *Pten* mutant reporter construct (*Pten*-Mut), we introduced mutations at the predicted m6A modification site of *Pten* and cloned it into the psiCHECK-2 luciferase reporter plasmid. Subsequently, AECII cells with repressed METTL3 expression and control cells transfected with shNC were separately transfected with Pten-WT and Pten-Mut constructs. Following a 48-hour incubation period, we assessed luciferase activity using the dual-luciferase reporter assay (Promega, Beijing, China).

### Quantification of m6A modifications

The TRIzol reagent (Life Technologies) was used to extract total RNA from lung tissue and AECII. Following the manufacturer’s protocol, we employed the m6A RNA Methylation Quantification Kit (Colorimetric) (Abcam ab185912) to quantify levels of m6A modification. Specifically, 200 ng of RNA was incubated in assay wells at 37 °C for 90 min. Subsequently, we introduced capture antibody, detection antibody, and enhancer solution into the well in a sequential manner. Finally, signal detection was performed by adding a color-developing solution, and the overall level of m6A modifications was measured from absorbance at 450 nm using the standard curve.

### Dot-blot assays

The TRIzol reagent (Life Technologies, MA, USA) was utilized to extract total RNA from lung tissue and AECII following the manufacturer’s instructions. The isolated mRNAs were subjected to denaturation using UV irradiation for a duration of 7 min and subsequently cooled on ice. Subsequently, these mRNAs were applied onto a nucleic acid transfer-optimized membrane (GE Healthcare, USA). The membrane underwent cross-linking through UV irradiation, followed by blocking with 5% non-fat milk and incubation with anti-m6A antibody overnight at 4 °C. Finally, visualization of the membrane was achieved using Immobilon Western Chemilum HRP Substrate (Merck Millipore, Germany).

### m6A-seq

Total RNA was extracted from AECII after METTL3 knockdown. The total RNA of each sample was quantified by the NanoDrop ND-1000 spectrophotometer, and the integrity of RNA was evaluated by the Bioanalyzer 2100 or Mops electrophoresis. Following Arraystar’s standard protocol, sample preparation and microarray hybridization were executed. Concisely, the total RNAs underwent immunoprecipitation with anti-N6-methyladenosine (m6A) antibody. RNAs bearing this modification extracted from the immunoprecipitated magnetic beads were designated as “IP”. Conversely, the unmodified RNAs retrieved from the supernatant were designated as “Sup”. Following the Arraystar RNA Labeling protocol, the “IP” and “Sup” RNAs were individually labeled with Cy5 and Cy3 to generate cRNAs in separate reactions. These cRNAs were subsequently combined and hybridized onto the Arraystar Mouse mRNA&lncRNA Epitranscriptomic Microarray (8 × 60 K, Arraystar). After the slides were thoroughly washed, the arrays were scanned using two-color channels on an Agilent Scanner G2505C.

### m6A-seq data analyses

The Agilent Feature Extraction software, version 11.0.1.1, was utilized for the analysis of the array images obtained. For IP (immunoprecipitated, Cy5-labelled) and Sup (supernatant, Cy3-labelled), their raw intensities were subjected to normalization using the average log2-scaled Spike-in RNA intensities. Following the Spike-in normalization process, probe signals that exhibited either Present (P) or Marginal (M) Quality Control (QC) flags in a minimum of three out of the six samples were selected for subsequent “m6A quantity” analysis. The “m6A quantity” was determined to calculating the amount of m6A methylation based on the normalized intensities of the IP (Cy5-labelled). The distinction in RNAs that were differentially m6A-methylated between the two comparison groups was discerned through a filtration process that applied thresholds for fold change and statistical significance, as denoted by p-value. Hierarchical clustering was conducted to demonstrate the distinguishable m6A methylation patterns among the samples.

### Measurement of *Pten m*RNA immunoprecipitation assay

“To quantify the interaction between METTL3 and Pten mRNA, we conducted an immunoprecipitation assay. Briefly, we incubated 1.5ug of anti-METTL3 (Abcam, ab195352) or anti-IgG (Cell Signaling Technology) antibody with protein A/G magnetic beads overnight at 4 ℃ to form conjugates. Subsequently, a mixture of 100 µg total RNA and the antibody was added to the immunoprecipitation buffer containing RNase and protease inhibitors. Following this, elution buffer was used to extract the RNA which was then purified using TRIzol for subsequent qRT-PCR analysis.

### m6A RNA immunoprecipitation assay

The MeRIP assay for m6A RNA immunoprecipitation was conducted using the Magna MeRIP m6A kit (Millipore, USA) as per the manufacturer’s instructions. Briefly, we incubated 3 µg of an anti-m6A antibody (Abcam) with protein A/G magnetic beads overnight at 4 °C to form conjugates. Subsequently, the antibody-conjugated beads were mixed with RNase and protease inhibitors in the immunoprecipitation buffer along with the RNA of interest. The resulting precipitate was subjected to digestion using proteinase K buffer, followed by isolation of co-immunoprecipitated RNA for qRT-PCR and RT-PCR analysis.

### mRNA stability

The AECII cells were cultured in 96-well plates with a density of 5 × 10^5^ cells/ml and subjected to various treatments. Subsequently, the cells were treated with actinomycin D (Act-D, cat. #A9415, Sigma) at a concentration of 5 µg/ml. Following incubation for the required durations, the collected cells were utilized for RNA analysis using qRT-PCR. The expression data obtained was normalized based on the β-actin mRNA levels.

### mRNA translation efficiency

After different treatment, nuclear extracts of AECII were extracted and sonicated. After a pre-incubation period of 2 h at 4 °C with protein A beads, the sonicated nuclear extracts were subjected to an overnight incubation at 4 °C with 20 µl of protein A beads and anti-eIF3A antibodies (anti-IgG reaction served as the experimental control). The solution underwent a washing process using the RIP buffer, and the precipitation was resuspended by PBS. After incubation with DNase, the Proteinase KRNA was extracted from the beads, subjected to purification using a Qiagen RNeasy column, and subsequently underwent reverse transcription. Real-time quantitative PCR was employed to identify the enrichment of Pten mRNA.

### ELISA

The levels of IL-6 and TNF-α were determined using ELISA (cat. Nos. ml063159 and ml002095, Meilian Biotech, China) by measuring the absorbance at 450 nm in accordance with the manufacturer’s provided guidelines.

### Whole-body plethysmography

The mice were subjected to weighing and subsequently introduced into a whole-body plethysmography apparatus (WBP-4MR, TOW, China) for an adaptation period of 5–10 min. The commencement of the experiment occurred once the mouse’s condition reached a stable state. Four mice could be simultaneously recorded in different colors.

### Bioinformatics analysis

SRAMP (http://www.cuilab.cn/sramp/) was utilized for the prediction of m6A modification patterns in the target mRNAs. PRIdictor (http://www.rna-society.org/rnainter/PRIdictor.html) and RPISeq were employed to forecast protein binding sites on the target mRNA. RMbase v2.0 (http://rna.sysu.edu.cn/rmbase/) was used to identify the primary mRNA m6A motifs that interacted with METTL3.

### Statistical analysis

The statistical analysis was performed using GraphPad Prism 8 Software (GraphPad Prism, CA, USA). The mean ± standard deviation was used to present the data. Independent sample t-test was employed to compare differences between two groups, while one-way analysis of variance (ANOVA) followed by Tukey’s test was utilized for comparisons among multiple groups. Statistical significance of group effects was determined at *P* < 0.05 level.

## Results

### METTL3 expression and m6A modification were elevated in the lung tissues in the ALI mice

**Fig. 1:**
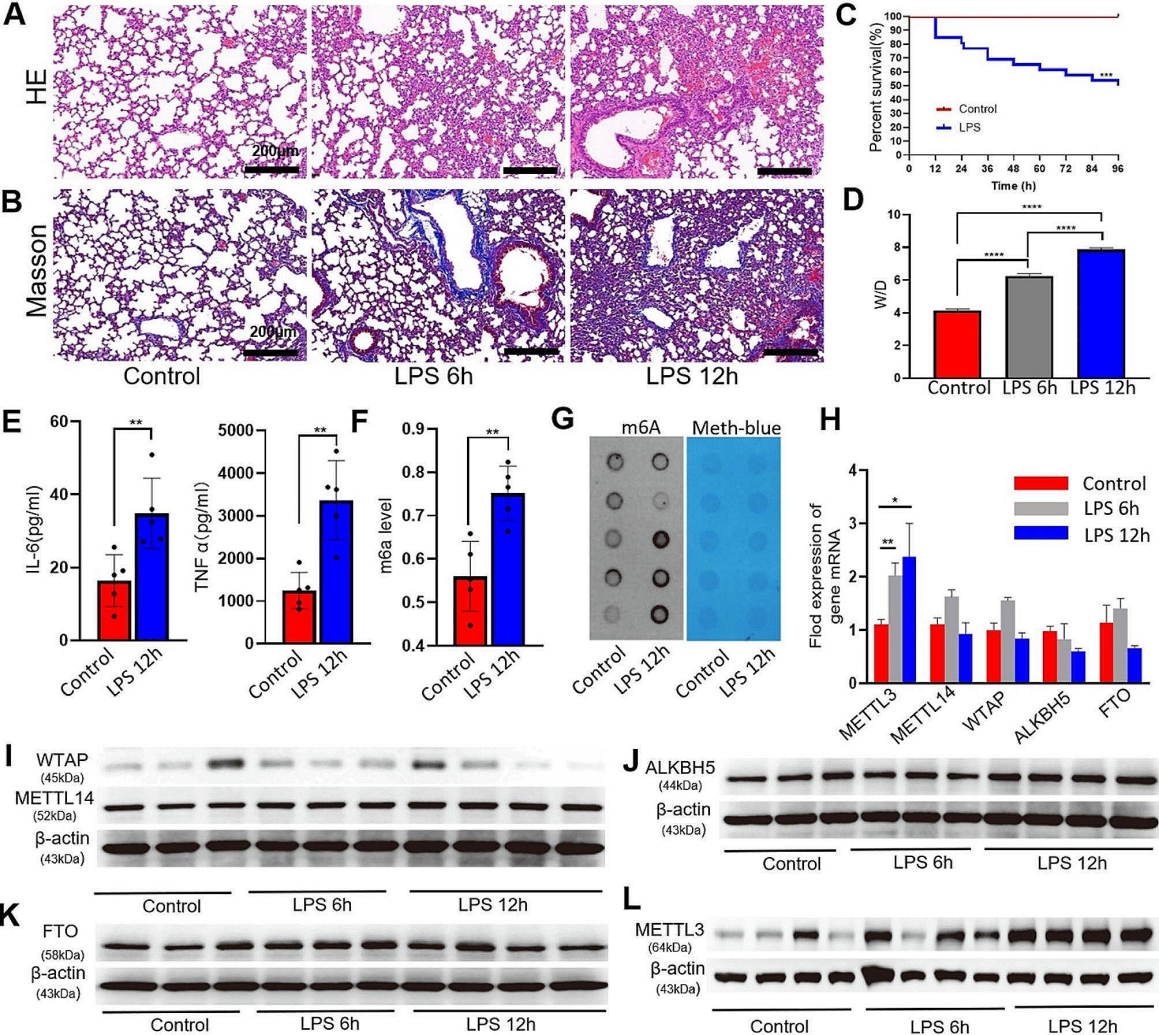
Up-regulation of the number of m6A-modified loci and increased expression of METTL3 were observed in lung tissue following ALI induction. In this study, we examined various morphological and molecular parameters in the lung tissue of rats treated with either a sham procedure or LPS at 6 and 12 h post-treatment (**A, B**) Hematoxylin-eosin and Masson’s trichrome staining. (**C**) The survival rates were monitored for a duration of 96 h following exposure to LPS (*n* = 20/group). (**D**) Pulmonary edema severity was evaluated by determining the wet to dry ratio (W/D ratio). (**E**) ELISA of IL-6 and TNF-α concentrations. Total m6A modification levels were detected by colorimetric (**F**) and dot blot experiment (**G**). Methylene blue (MB) staining served as a loading control (blue stripe). (**H**) qPCR analyses of mRNA expression levels of the m6A “writers” (METTL14, METTL3, and WTAP) and “erasers” (ALKBH5 and FTO) 6 h and 12 h after LPS administration. (**I–L**) Protein expression of m6A “writers” (METTL14, METTL3, and WTAP) and “erasers” (ALKBH5 and FTO) in ALI. Statistical significance of differences is denoted as follows: ns, not significant; *P < 0.05; **P < 0.01; ***P < 0.001; ****P < 0.0001

The ALI model was induced in mice through the intraperitoneal injection of LPS. Examination using hematoxylin/eosin staining revealed a noticeable thickening of the alveolar wall, distinct infiltration of inflammatory cells, and presence of alveolar hemorrhage within the initial 12 h following LPS administration (Fig. 1A). Additionally, Masson’s trichrome staining demonstrated an increase in collagen fibers within the lung interstitium (Fig. 1B). The edema of lung was assessed by the W/D ratio, which increased significantly 12 h after LPS treatment (*P* < 0.0001, Fig. 1D). The survival rate of the LPS-treated mice was significantly reduced (Fig. 1C), while the levels of inflammatory cytokines, TNF-α and IL-6, exhibited a gradual increase over the time compared to those control animals (Fig. 1E). The rate of m6A modifications in the lung tissues exhibited a significant and rapid increase within 12 h after LPS administration (Fig. 1F, G)We further detected the changes of METTL14, WTAP, FTO and ALKBH5 treated with LPS for 6 h and 12 h. The expression of METTL3 significantly increased at 6 h and 12 h.in mRNA (Fig. 1H) and protein levels (Fig. 1I-L) in lung tissues.

### Methyltransferases inhibitor alleviated lung injury and improved lung functions in the ALI mice

**Fig. 2:**
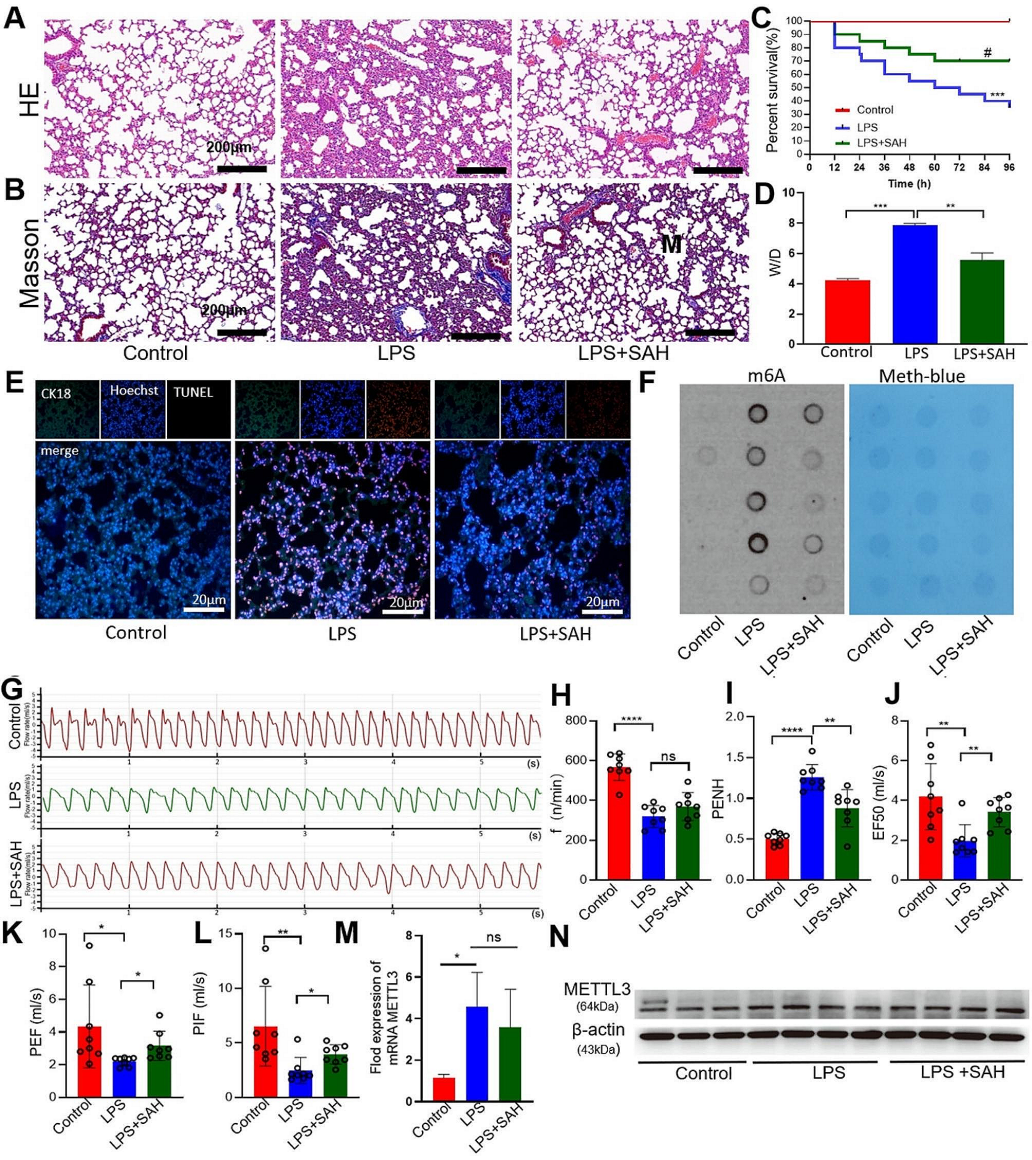
Treatment with SAH reduce lung injury and improve lung function. (**A, B**) Hematoxylin-eosin and Masson’s trichome staining of rat lung tissue (Control, LPS, and LPS + SAH treatment groups). (**C**)The survival rates were observed during 96 h (Control, LPS, and LPS + SAH treatment groups) (*n* = 20/group). (**D**) The evaluation of pulmonary edema severity was conducted using the wet to dry ratio (W/D ratio). (Control, LPS, and LPS + SAH treatment groups). (**E**) The presence of apoptotic cells was detected in the tissues. (**F**) Total m6A modification levels were detected by dot blot experiment. Methylene blue (MB) staining served as a loading control (blue stripe). (**G**-**L**) Respiration waveforms of mice and lung function indexes of airway obstruction were evaluated between groups (Control, LPS, and LPS + SAH treatment groups). (*n* = 8). (**M**) qPCR analyses of mRNA expression levels of the METTL3 (Control, LPS, and LPS + SAH treatment groups). (**N**) METTL3 protein expression was analyzed using Western blotting (Control, LPS, and LPS + SAH treatment groups) .The data were normalized based on the expression level of β-actin. Statistical significance of differences is presented as follows: #*p* < 0.05 vs. LPS alone group *P < 0.05; **P < 0.01; ***P < 0.001; ****P < 0.0001

To conduct additional experiments on the role of m6A modification in acute lung injury, we administered S-adenosylhomocysteine (SAH), a methyltransferases inhibitor, via intraperitoneal injection and conducted whole-body plethysmography. The treatment with SAH effectively reduced the histological damages in the lungs caused by LPS. (Fig. 2A, B). The W/D ratio was reduced and the survival rate of animals with ALI was increased by SAH treatment. (Fig. 2C, D). To investigate the potential impact of methylated modification on apoptosis in ALI mice induced by LPS, we conducted a TUNEL staining assay to examine apoptotic alveolar cells following SAH treatment. The findings revealed a significant elevation in the number of TUNEL-positive cells as a result of LPS exposure, but this increase was obviously inhibited by SAH (Fig. 2E), and tissue RNA m6A modification levels were significantly reduced after SAH intraperitoneal injection than ALI group (Fig. 2F). We further examined the changes of lung function, and found that an elevation in airway resistance PENH and EF50 among the LPS group, and the intervention by SAH resulted in the reversal of this change. As indicators of airway conductivity index, PEF and PIF, which reflect the conductivity index, were significantly decreased after SAH-treatment. (Fig. 2G-K). We detected the expression of METTL3 in lung tissues treated with SAH by qPCR and WB. Figure 2 (M, N) showed that SAH had no significant statistical difference in the expression of METTL3. Consider that SAH inhibited the overall methylation level rather than acting on the methyltransferase METTL3. S-adenosylmethionine (SAM) is the principal biological methyl donor synthesized in all mammalian cells but most abundantly in the liver. SAH is a metabolite of SAM during the methyl-transfer reaction. As SAH and SAM similar in structure, SAH consider as strong inhibitory effect on all SAM dependent methyltransferases. In our study, we analysis glutamic-pyruvic transaminase (GPT) and glutamic oxaloacetic transaminase (GOT) in serum. Result showed that GPT and GOT significant increased in LPS-treated mice serum compared with control group, while no obvious difference between SAH administer and LPS-treated (Supplementary Fig. 2). While the impact of LPS indued in liver injury cannot be excluded, which is necessary for future exploration. In view of the importance function of ACEII, we isolated and cultured the primary ACEII in vitro to further explore the mechanism (Supplementary Fig. 2).

### In vitro, LPS was found to augment the level of m6A modifications and METTL3 expression in AECII

**Fig. 3:**
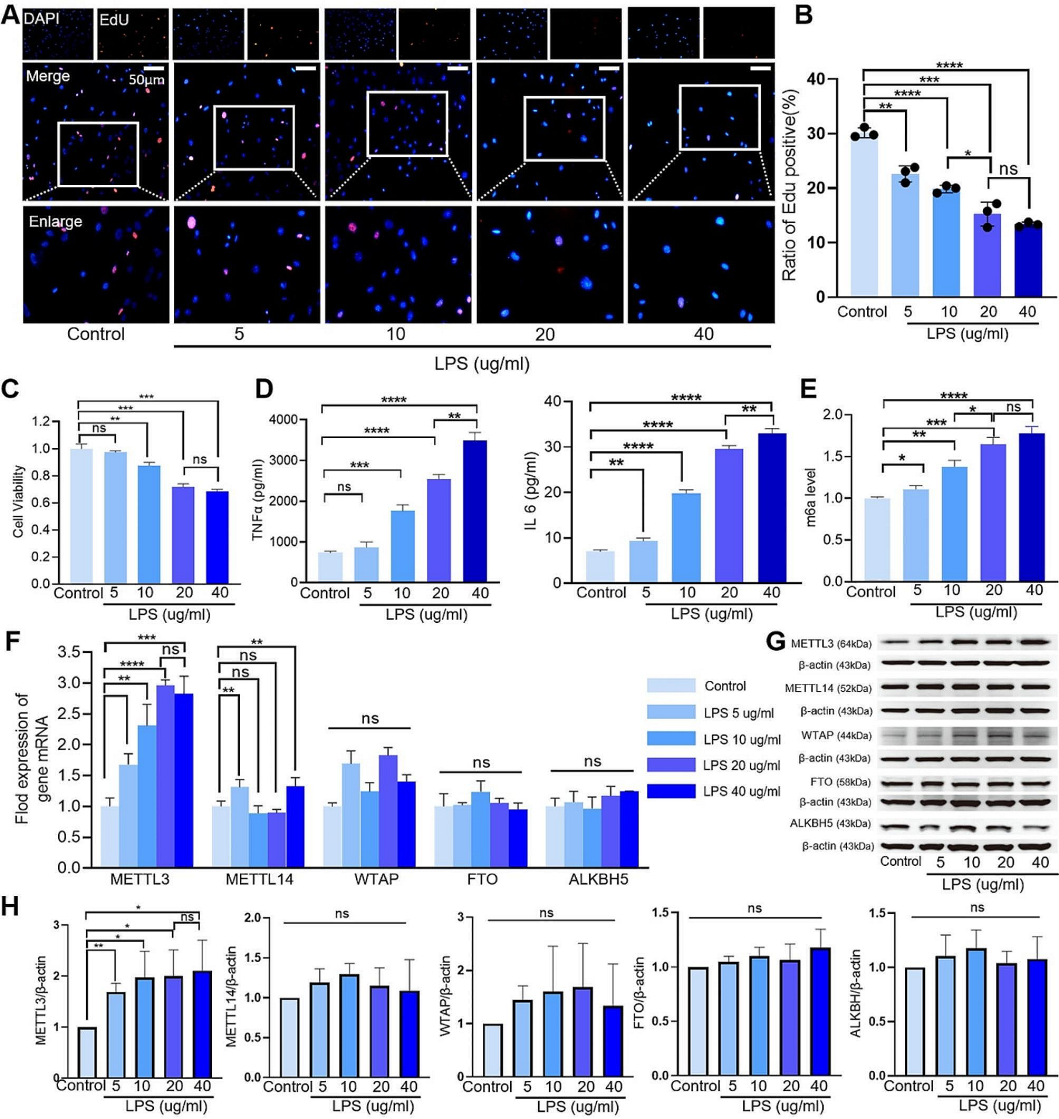
Treatment with LPS leads to a reduction in the proliferation and viability of AECII, an increase in the level of m6A-modified loci, and an upregulation of METTL3 expression. (**A-B**) The EdU assay demonstrates a decrease in AECII proliferation following treatment with LPS at concentrations ranging from5–40 µg/ml. (**C**) The CCK-8 assay reveals a decline in the viability of LPS-treated AECII. (**D**) ELISA analysis shows elevated IL-6 and TNF-α concentrations in AECII after exposure to LPS. (**E**) Enhanced total m6A modification levels observed in AECII following LPS treatment. (**F**) qPCR analyses of mRNA expression levels of m6A “writers” (METTL14, METTL3, and WTAP) and “erasers” (FTO and ALKBH5) in AECII. (**G–H**) Western blot analyses of protein expression levels of m6A “writers” (METTL14, METTL3 and WTAP) and “erasers” (ALKBH5 and FTO) in LPS-treated AECII. The data were normalized based on the expression level of β-actin and GAPDH. Statistical significance of differences is presented as follows: ns, not significant; *P < 0.05; **P < 0.01; ***P < 0.001; ****P < 0.0001

Various concentrations of LPS (5, 10, 20, and 40 µg/ml) were administered to primary AECII cultures. After 12 h treatment, LPS dose-dependently reduced the proliferation (Fig. 3A, B) and viability (Fig. 3C) of AECII, increased the inflammatory cytokines (Fig. 3D), and enhanced the m6A modification and the METTL3 expression (Fig. 3E). Although LPS at 5–10 µg/ml decreased the viability and proliferation of AECII in varying degrees, the reduction reached a peak with LPS at 20 µg/ml. Therefore, in the following study for mechanism investigation, we chose 20 µg/ml as the optimal concentration of LPS. Notably, while the m6A modification of AECII exhibited a dose-dependent increase in response to LPS stimulation in vitro, it was specifically METTL3 rather than other “writers” of m6A modification increased accordingly (Fig. 3E, F). The “erasers” of m6A modification, FTO and ALKBH5, were not changed in either mRNA or protein level after LPS treatment (Fig. 3F-H). The increment of m6A modification of AECII also arrived at the peak when exposed to the LPS at 20 µg/ml.

### Downregulation of METTL3 alleviated the damages of AECII and improved the pulmonary functions after LPS insults

**Fig. 4:**
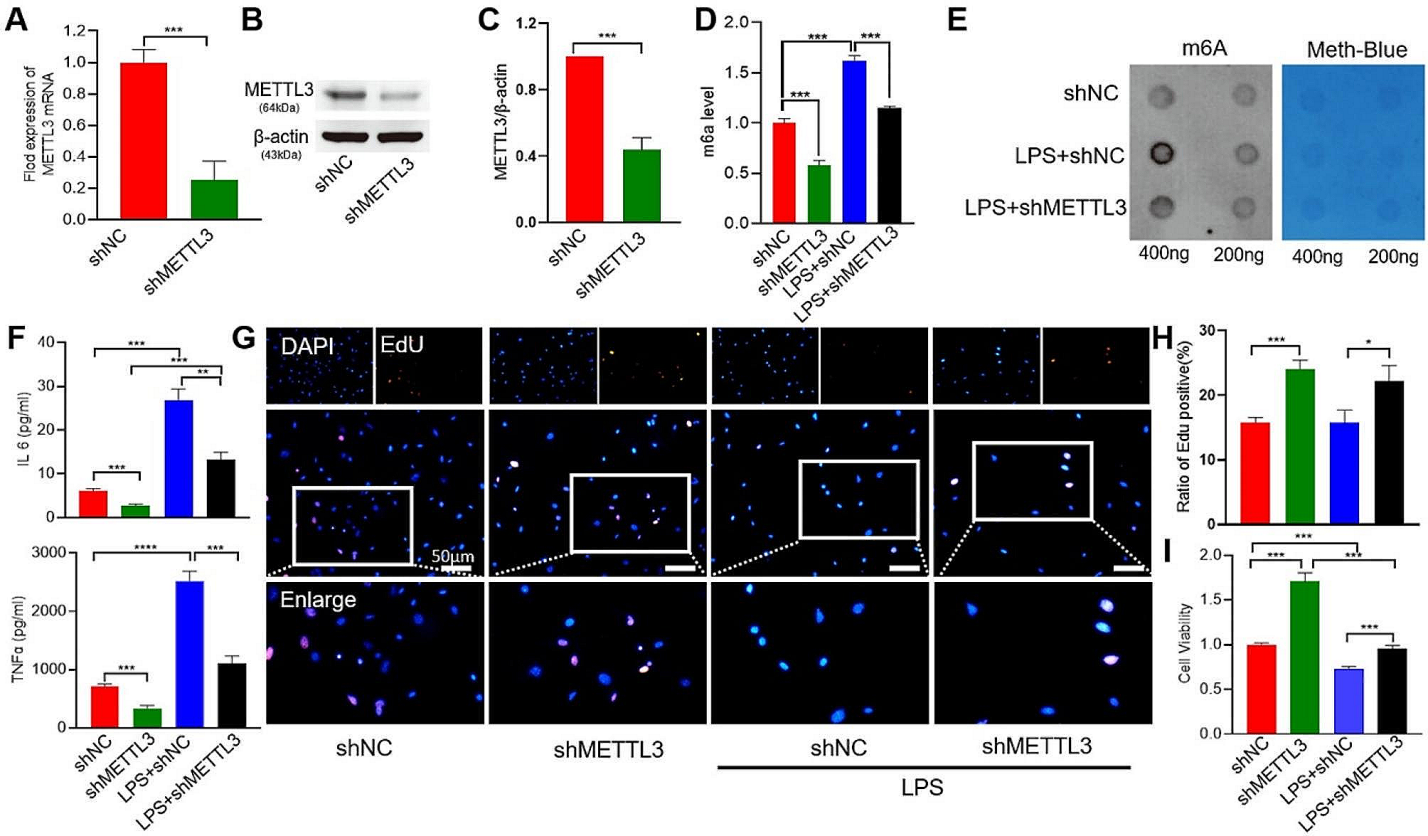
In vitro and in vivo, the expression of METTL3 in AECII is enhanced by exposure to LPS. Several molecular parameters were assessed in AECII that were exposed to LPS or LPS + *shMettl3*. (**A–C**) qPCR and western blot analyses of METTL3 knockdown efficiency. Expression levels of β-actin mRNA and protein served as controls. Total m6A modification levels in AECII were categorized into Control, LPS, and LPS + shMETTL3 groups. (**D**) m6A RNA Methylation Quantification Kit assay (**E**) Dot-blot assay (**F**) ELISA of IL-6 and TNF-α concentrations (**G–H**) Proliferation of AECII analyzed using the EdU assay. (**I**) Viability of AECII analyzed using the CCK-8 assay. Statistical significance of differences is denoted as follows: #*p* < 0.05 vs. LPS alone group *P < 0.05; **P < 0.01; ***P < 0.001; ****P < 0.0001

In order to examine how METTL3 regulates the damages of AECII in LPS mice, we knocked-down METTL3 of AECII by using shRNAs. The efficacy of shRNA knockdown was validated by means of qRT-PCR and western blotting (Fig. 4A-C). Consequently, the level of m6A in the AECII was markedly decreased compared to the shMETTL3 intervention with LPS treatment (Fig. 4D, E). The knockdown of METTL3 resulted in a significant reduction in the levels of inflammatory factors IL-6 and TNF-α (Fig. 4F), while also leading to a notable increase in the proliferation and viability of LPS-treated AECII with shMETTL3. (Fig. 4G-I).

### METTL3-mediated m6A methylation modulated the damages of LPS-treated AECII via facilitating the translation and stability of PTEN

**Fig. 5:**
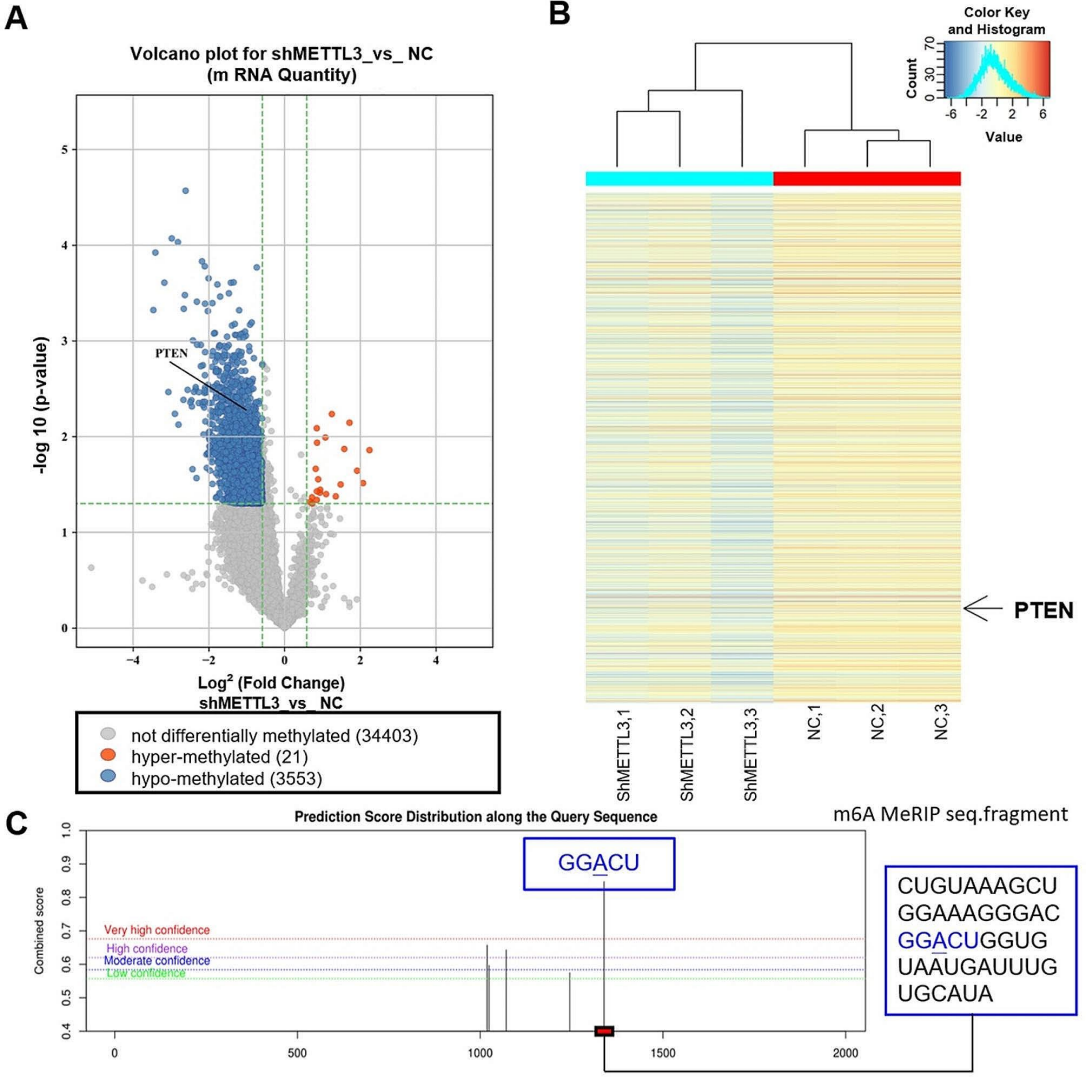
Regulation of *Pten* mRNA expression in AECII is influenced by METTL3-mediated m6A modifications. (**A**) Volcano plot analysis of 21 upregulated and 3 553 downregulated mRNAs in AECII transfected with *shMettl3* (*P* < 0.05 vs. negative control). (**B**) Cluster analysis of altered m6A-methylated mRNAs in AECII transfected with *shMettl3* (*P* < 0.05 vs. negative control). (**C**) Predictive analysis using the SRAMP database revealed the presence of m6A loci on mouse *Pten* mRNA

Therefore, our aim is to investigate the target gene of METTL3-mediated m6A modification in AECII. We utilized the m6A epitranscriptomic microarray analysis to assess changes in mRNA modifications following knockdown of METTL3 in AECII. Out of a total of 3,574 mRNAs analyzed, alterations in m6A methylation were observed for 3,553 mRNAs, including PTEN which exhibited hypomethylation. (Fig. 5A, B). Next, by using the SRAMP database, the m6A loci at *Pten* mRNA was successfully predicted (Fig. 5C). In this study, we tried to demonstrate that PTEN contains methylation modification sites, which may be the downstream target of METTL3.

**Fig. 6:**
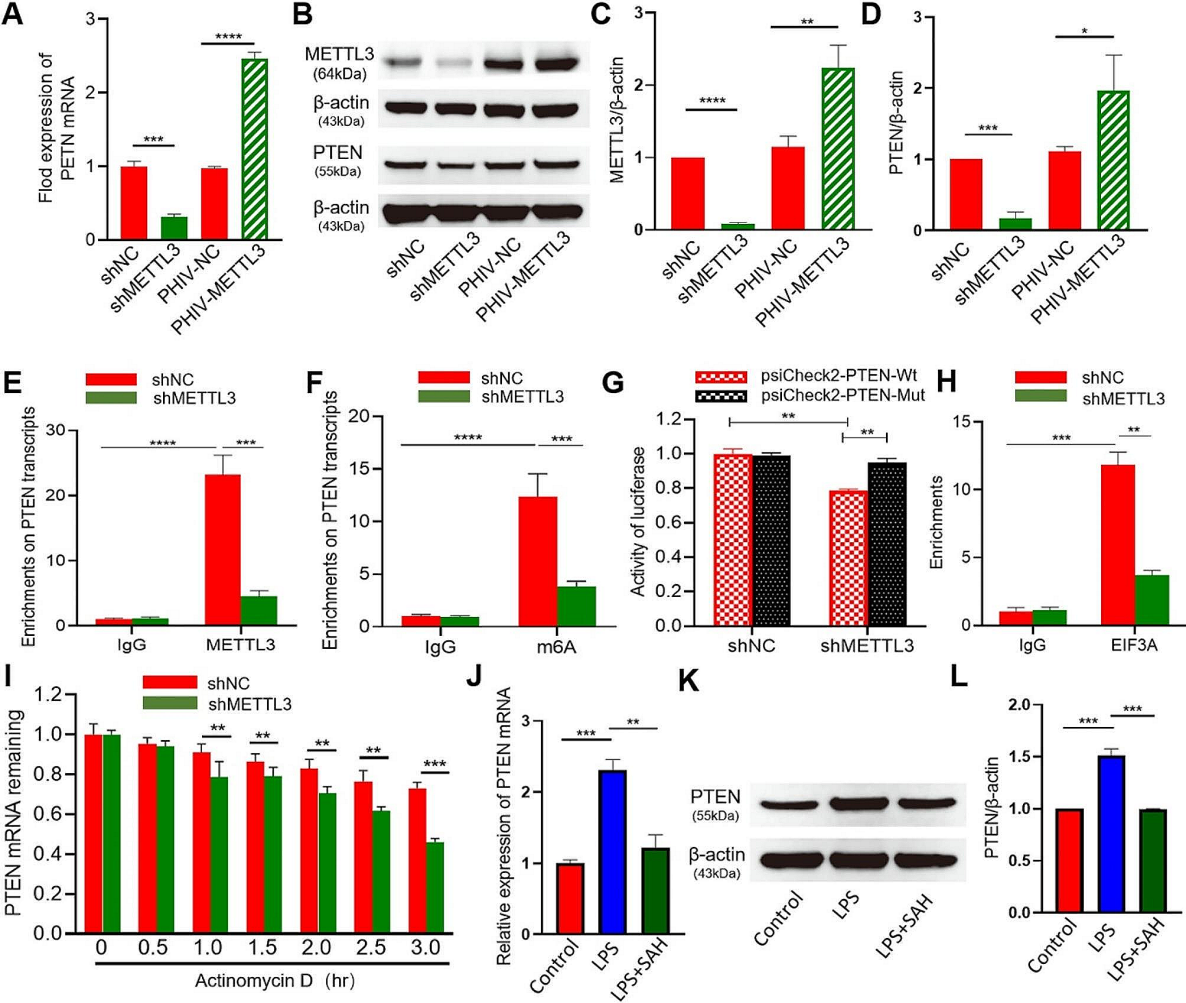
METTL3-dependent m6A modifications regulate the stability and translation efficiency of *Pten* mRNA in AECII. (**A, B**) Analysis of the efficiency of METTL3 overexpression using Western blotting. (**A, C**) Revealing PTEN protein expression levels through western blot analysis following knockdown or overexpression of METTL3 in AECII. (**D**) Revealing PTEN gene levels in AECII through qPCR analysis following knockdown or overexpression of METTL3. (**E**) Analysis of the interaction between METTL3 and Pten transcripts in AECII using METTL3-RIP and gene-specific qPCR. (**F**) Detection of m6A modifications in *Pten* transcripts following knockdown of METTL3 using m6A-RIP and gene-specific qPCR analysis. (**G**) Wild-type and mutant binding sites of METTL3 in the 3′-UTR of *Pten* mRNA. (**H**) Analysis of *Pten* mRNA half-life using qPCR reveals a reduction in Pten mRNA stability following knockdown of METTL3. (**I**) Revealing the translation efficiency of *Pten* through gene-specific PCR coupled with immunoprecipitation analysis. (**J–L**) Revealing the levels of *Pten* gene and protein expression through qPCR and western blotting post SAH treatment. Statistical significance of differences is presented as follows: *P < 0.05; **P < 0.01; ***P < 0.001; ****P < 0.0001

**Fig. 7:**
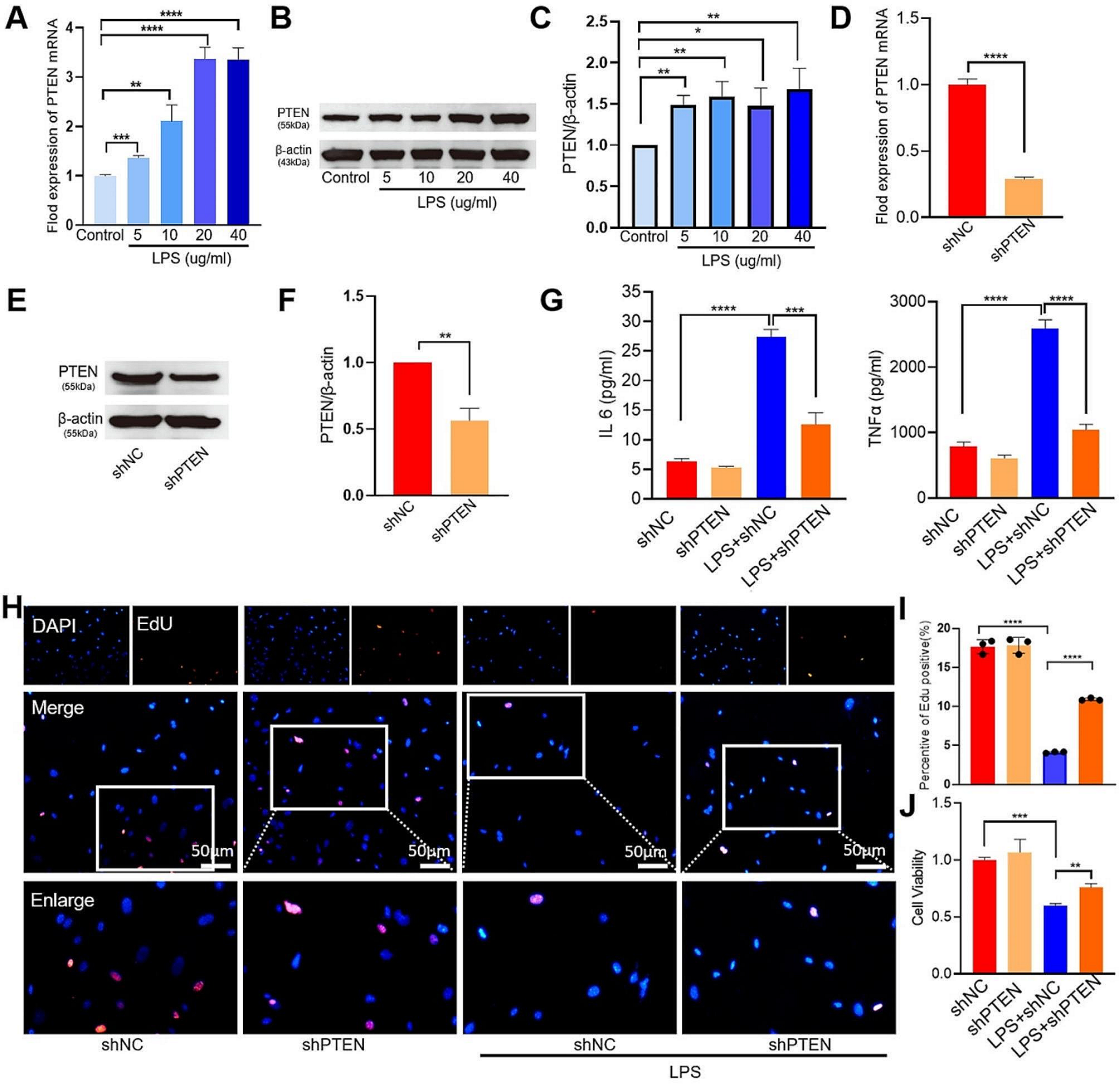
PTEN regulates AECII proliferation and viability. (**A-C**) The expression levels of PTEN gene and protein in AECII samples treated with LPS at concentrations ranging from 5 to 40 µg/ml were assessed using qPCR and western blot analysis. All expression data were standardized to β-actin mRNA and protein levels as reference controls. (**D-F**) The efficiency of PTEN knockdown in AECII was assessed using qPCR and western blot analyses. The data were normalized with respect to the expression levels of β-actin mRNA and protein, which were used as internal controls. (**G**) ELISA of IL-6 and TNF-α levels in AECII treated with LPS or LPS + *shPten.* (**H–I**) Proliferation of AECII treated with LPS or LPS + *shPten* evaluated by the EdU assay. (**J**) Viability of AECII treated with LPS or LPS + *shPten* evaluated by the CCK-8 assay. Statistical significance of differences is denoted as follows: *P < 0.05; **P < 0.01; ***P < 0.001; ****P < 0.0001

Previous reports have confirmed PTEN is crucially involved in the regulation of lung cell migration, proliferation and viability in lungs [15], we further explore its function in the AECII. We overexpressed the METTL3 by transfecting AECII with full gene sequence METTL3 plasmid(GentleGen)and knocked down METTL3 by transfecting shMETTL3 plasmid. The efficacy of overexpression and knockdown was confirmed by qRT-PCR and western blotting (Fig. 6A-D). Co-immunoprecipitation showed *Pten* mRNA was fully m6A-modified, and PTEN abundance was markedly decreased upon METTL3 knockdown (Fig. 6E, F). To investigate the specific interaction between METTL3 and *Pten* mRNA, we generated dual luciferase reporters containing either the unaltered or modified 3′-UTR of *Pten*. Knockdown of METTL3 resulted in a significant reduction in m6A modification levels in the mutant *Pten* 3′-UTR (Fig. 6G). To assess the translation efficiency of *Pten* mRNA, we conducted immunoprecipitation gene-specific PCR. The findings revealed a significant reduction in the translation efficiency of *Pten* mRNA following knockdown of METTL3 (Fig. 6H). The *Pten* mRNA degradation was significantly enhanced upon METTL3 knockdown, as demonstrated by the RNA stability assay (Fig. 6I). Our result showed METTL3 mediated the m6A methylation via facilitating the translation and stability of PTEN, we further intent to investigate whether PTEN could reduce AECII injury. As we expected, the expression of PTEN significantly increased in lung tissues, whereas the level of PTEN were obviously inhibited after SAH injection (Fig. 6J-L). Furthermore, in the LPS-treated AECII in vitro, PTEN expression was increased (Fig. 7A-C). When PTEN was knocked down (Fig. 7D-F), the proliferation and viability of AECII treated with LPS were clearly increased (Fig. 7H-J), and the levels of IL-6 and TNF-α exhibited significant suppression (*P*<0.001, Fig. 7G).

### METTL3 inhibited the viability and proliferation of LPS-insulted AECII via PTEN/ PI3K/AKT pathway

**Fig. 8:**
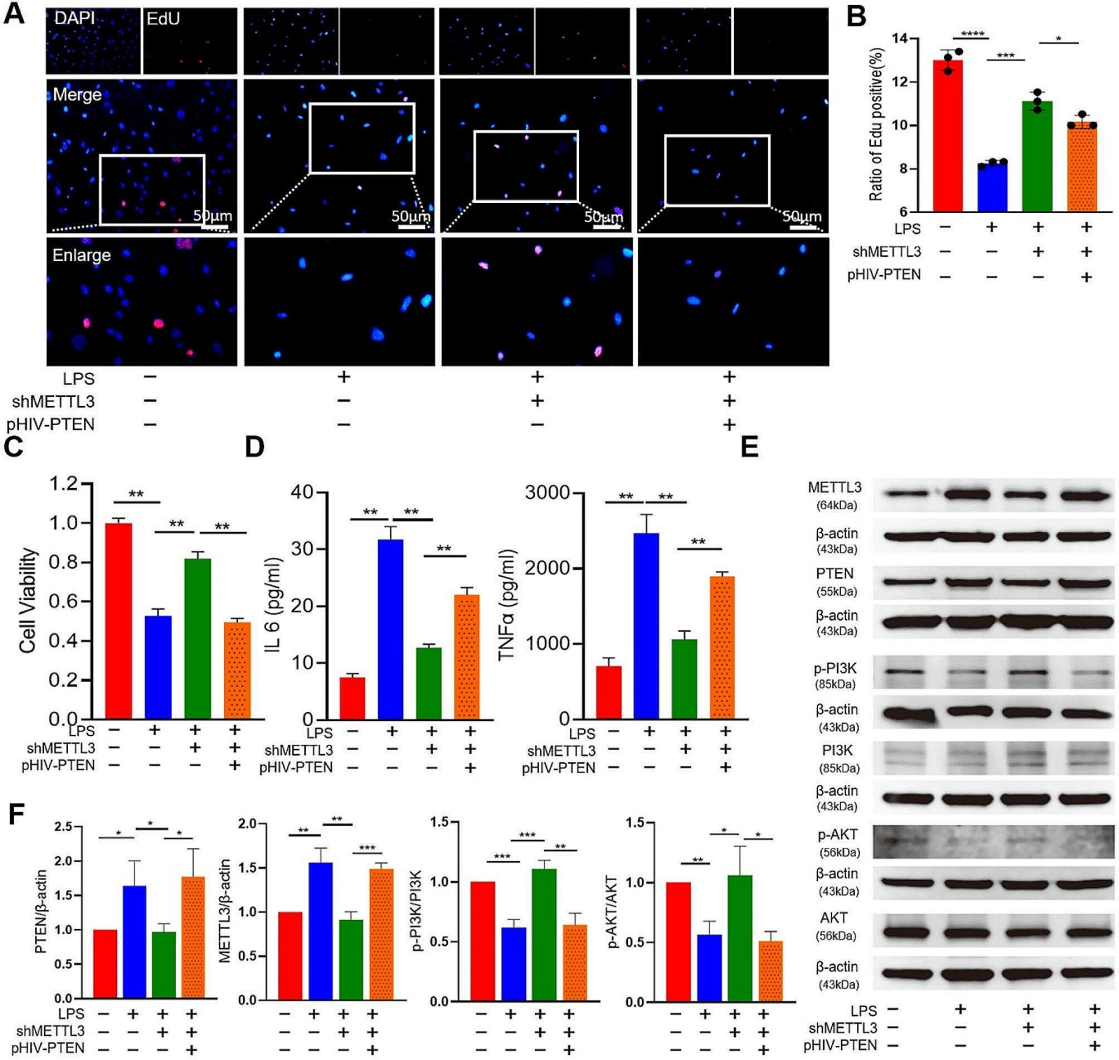
METTL3-dependent m6A modifications influence the PETN/PI3K/AKT pathway signaling in AECII. Proliferation, viability, and several molecular properties of AECII treated with 20 µg/ml LPS, *shMettl3*, and *pHIV*-*Pten* were studied. (**A, B**) Proliferation of AECII analyzed by the EdU assay. (**C**) CCK-8 assay analysis of AECII viability. (**D**) ELISA of IL-6 and TNF-α levels. (**E–F**) PI3K, p-AKT, and AKT expression levels in AECII evaluated by western blotting. Expression level of β-actin served as control. Statistical significance of differences is indicated as follows: *P < 0.05; **P < 0.01; ***P < 0.001; ****P < 0.0001

To investigate the impact of PTEN on AECII damages in the ALI model, we transfected the AECII with plasmid to knockdown or overexpress PTEN. As shown in the Fig. 8A–D, METTL3 knockdown resulted in enhanced viability and proliferation of AECII, and reduced the inflammatory cytokines after LPS insults. In contrast, overexpression of PTEN in AECII reversed the protective effect of METTL3 knockdown, by reducing the alveolar viability and proliferation but also increasing the inflammation. PTEN is normally considered to regulate cell proliferation, apoptosis and inflammation through PI3K/AKT signal pathway [16, 17]. In our investigation, the levels of PI3K and p-AKT were found to be reduced in AECII treated with LPS. Moreover, when PTEN was overexpressed, it further diminished the levels of p-PI3K and p-AKT in AECII (Fig. 8E, F). Taken together, these findings suggest that PTEN exerts a suppressive effect on AECII differentiation by partially inhibiting the signaling pathway of PI3K/AKT.

## Discussion

**Fig. 9:**
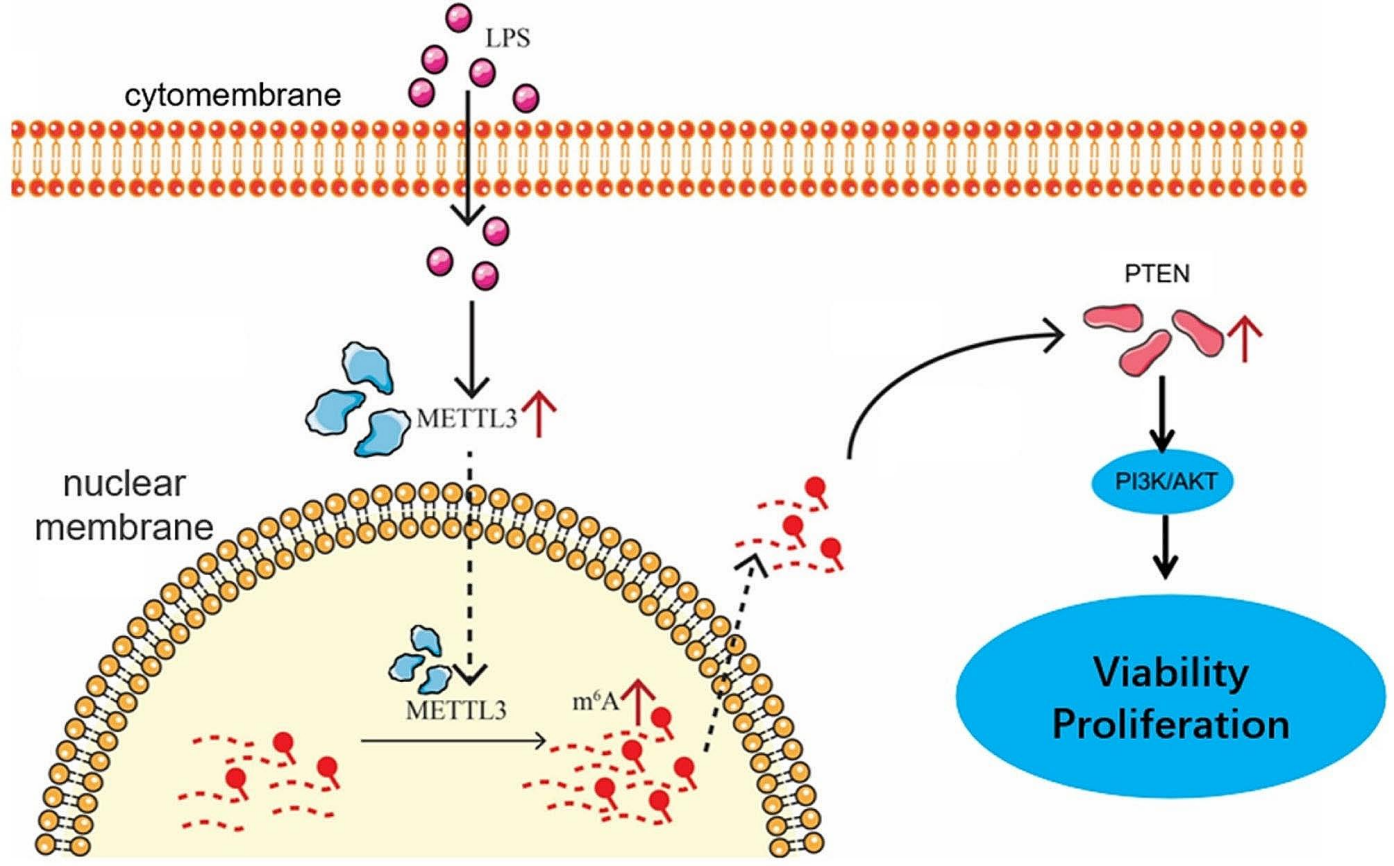
Model of theMETTL3-dependent m6A modifications influence the PETN/PI3K/AKT pathway signaling in AECII

Accumulating evidence has brought attention to the essential role of AECII in mediating alveolar epithelial recovery in lung injuries [18]. Repair of the alveolar epithelium after injury largely depends on the AECII proliferation and differentiation [19, 20]. In the current study, we identified that the increase of METTL3 improved the AECII proliferation and differentiation in the LPS-insulted mice via enhancing translation and stability of *Pten* by m6A modification (Fig. 9).

As the progenitor cells of alveolar cells, AECII can differentiate into AECI to repair damaged alveoli, and maintain the homeostasis and integrity of epithelial cells in ALI [21]. Prior research demonstrated that modulation of NLRP3 inflammasome activation in AECII mitigated the lung injury caused by LPS-induced ALI [22]. Foster production of cytokines interleukin-17 in AECII conferring a protective role in sepsis-induced ALI [23]. Our data confirmed that m6A methylation modification involves in LPS-induced AECII inflammatory.

In the past few years, there has been a significant increase in research focused on m6A, which is the most prevalent epigenetic modification of mRNA found in mammalian cells. Numerous studies have investigated the intricate mechanism behind m6A methylation, involving essential proteins such as methyltransferases and demethylases [24, 25]. In this investigation, we assessed the levels of three m6A methyltransferases and two demethylases in lung tissue affected by ALI as well as AECII stimulated with LPS in in vitro. The expression of METTL3 protein significantly increased at both 6 h and 12 h. Although the expression of METTL14, WTAP, FTO or ALKBH5 didn’t show that significant changes as METTL3. METTL3 is the only enzyme in the m6A writer complex that presents catalytic activity [26]. As reported in the recent study, the accumulation of reactive oxygen species (ROS) blocked upregulation of METTL3 in response to cytokines [27]. We separated AECII cells treated with LPS, and found the expression of METTL3 increased under the treatment of ROS inhibitors or NO inhibitors (Supplementary Fig. 3), suggesting that accumulation of NO and ROS may also downregulation and decreased enzymatic activity of METTL3 in AECII.

Our findings revealed a significant upregulation of METTL3 expression. Recent studies have demonstrated the involvement of m6A methylation in various conditions such as ALI, chronic obstructive pulmonary disease (COPD), pulmonary fibrosis, and lung cancer. [28, 29]. Zhang et al. discovered that METTL3 modification was triggered by neutrophil extracellular traps (NETs). In the mouse model of acute lung injury (ALI) lacking METTL3, there was a reduction in both lung damage and the levels of proinflammatory cytokines compared to the ALI mouse model with normal METTL3 expression [11]. METTL3 induces m6A methylation by promotes mitochondrial autophagy through the Pink1-Parkin pathway, leading to chemotherapy resistance in small cell lung cancer [30]. In addition, the modification of Trim59 by METTL3 was found to have a protective effect on endothelial injury in ARDS. [12]. Inhibition of METTL3 may potentially exhibit anti-apoptotic and protective effects against acute lung injury (ALI) by restoring the expression of neprilysin. Conversely, increasing the levels of METTL3 in mouse lungs demonstrated a preventive effect on LPS-induced ALI and decreased the synthesis of pro-inflammatory cytokines [31]. Previous studies, along with our own findings, propose that modulating m6A modification through adjustments in METTL3 levels could present a promising therapeutic approach for managing ALI. In our study, m6a level was increased in ALI lung injury, While the intraperitoneal administration of SAH, a transmethylase inhibitor, resulted in the improvement of lung function and reduction in alveolar exudation compared to the ALI group, it is widely acknowledged that AECII plays a crucial role in preserving the integrity of alveolar endothelium. However, the role of methylation modification AECII was not reported. Hence, it is imperative to conduct a comprehensive investigation on the additional molecular mechanisms associated with m6A methylation in AECII.

We observed an enhancement in the proliferation and viability of AECII upon METTL3 knockdown, accompanied by a decrease in inflammation. These findings shed light on the involvement of METTL3 in ALI regulation, indicating its potential as a promising therapeutic target. However, there was a lack of *in-vitro* study of the mechanism. In our study, we successfully isolated primary AECII and subjected them to LPS intervention. Our findings confirmed that the elevation of overall methylation levels following LPS treatment, coupled with METTL3 knockdown, effectively diminished the inhibitory effects of LPS on cell proliferation and viability, while concurrently reducing inflammation.

The utilization of m6A gene chip technology has facilitated the investigation into the biological function and regulation of this epigenetic modification. In comparison to previous methods such as m6A-seq, there has been a reduction in the RNA sample requirement. Additionally, it is now possible to observe modifications in lncRNA and other non-coding RNAs, thereby enhancing the identification of potential therapeutic targets. Consequently, we employed the m6A transcriptome microarray technique to assess changes in mRNA modifications following METTL3 knockdown in AECII cells. Our findings indicate that PTEN expression is regulated by METTL3. Significantly, the expression of PTEN was increased following LPS stimulation of AECII in vitro. PTEN primarily regulates various cellular processes such as cell proliferation, pyroptosis, apoptosis, adhesion, and migration by negatively modulating the PI3K/AKT signaling pathway. Additionally, it plays a crucial role in maintaining cell structure and facilitating signal transduction. Previous investigations have underscored the significance of PTEN in preserving the phenotypic integrity of airway epithelial cells [32–34]. In this study, enrichment experiments demonstrated that *Pten* mRNA recruited an m6A methyltransferase enzyme, resulting in increased levels of m6A methylation on *Pten*. Additionally, knockdown of METTL3 significantly reduced the levels of m6A methylation, stability, and translation efficiency of *Pten* mRNA. Consequently, this led to a decrease in PTEN protein expression. The crucial role played by PTEN in regulating cell motility, proliferation, and angiogenesis cannot be underestimated. As a well-known tumor suppressor gene, PTEN primarily modulates the PI3K/AKT signaling pathway under normal physiological conditions. He et al. confirmed m6A mRNA methylation participate in regulating endometrial cancer and identify m6A methylation as a regulator of AKT signal. Reductions in m6A methylation decreased expression of the negative AKT regulator PHLPP2 and increased expression of the positive AKT regulator mTORC2 [35]. Depletion of m6A levels in EndoC-βH1 induces cell-cycle arrest and impairs insulin secretion by decreasing AKT phosphorylation and PDX1 protein levels [36]. In our study, downregulation of METTL3 and overexpression of PTEN resulted in decreased activation of the PI3K/AKT signaling cascade. Thus, we have uncovered a novel mechanism involving the METTL3/PTEN/ PI3K/AKT signaling axis through m6A modification in AECII cells treated with LPS.

Some limitations are involved in our study. Although we successfully inhibited methylation level in vivo, the AECII-specific METTL3 knockout is still lacking. Besides, our tissue samples were taken after 12 h intraperitoneal injection of LPS, which was just at the earliest inflammation.

## Conclusion

Our investigation revealed an increase in the expression of METTL3 in AECII treated with LPS, leading to enhanced m6A methylation levels on Pten mRNA. Consequently, this modification improved the stability and efficiency of protein translation. PTEN plays a crucial role in regulating the PI3K/AKT signaling pathway, thereby controlling AECII proliferation and viability during ALI. In summary, these findings significantly contribute to our comprehension of ALI’s mechanism and identify a potential therapeutic target for managing this severe condition.

### Electronic supplementary material

Below is the link to the electronic supplementary material.

Supplementary Material 1

Supplementary Material 2

Supplementary Material 3

## Data Availability

No datasets were generated or analysed during the current study.

## Abbreviations

ALI: Acute lung injury
m6A: N6-methylation of adenosine
AECII: Alveolar type II cells
METTL3: Methyltransferase-like 3
Pten: Phosphatase and tensin homolog
METTL14: Methyltransferase-like 14
WTAP WT1: Associated protein
FTO: Fat mass and obesity-associated protein
ALKBH5 and alkB: Homolog 5
PI3K: Phosphoinositide-3 kinase
SAH: S-adenosylhomocysteine
TNF: Tumor necrosis factor
PENH: Enhanced pause
