# Supplementary figures and images for: METTL3-m6A methylation inhibits the proliferation and viability of type II alveolar epithelial cells in acute lung injury by enhancing the stability and translation efficiency of Pten mRNA
Source: Respir Res. 2024 Jul 15;25:276. doi: 10.1186/s12931-024-02894-z (PMC11251256; doi:10.1186/s12931-024-02894-z)

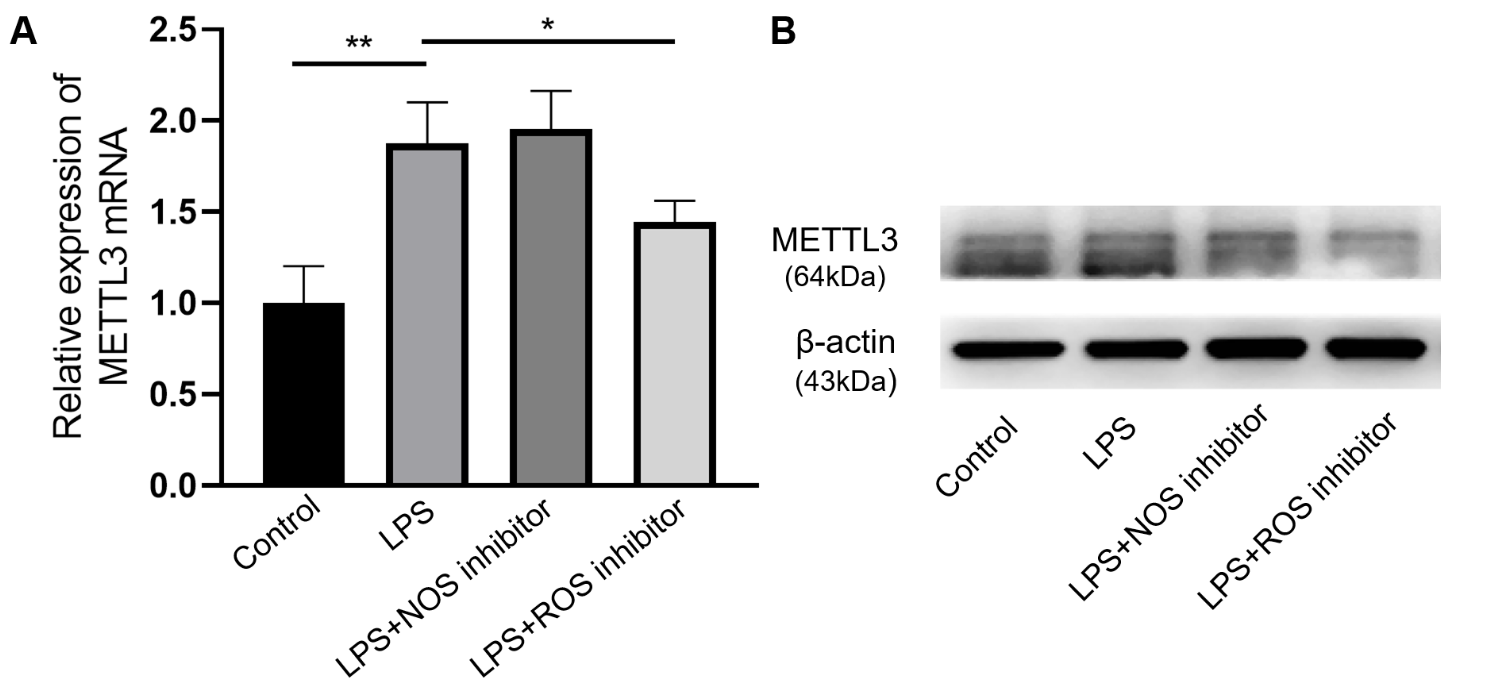

Supplement: Supplementary file 1 — Supplementary Material 1 [file 12931_2024_2894_MOESM1_ESM.tif]

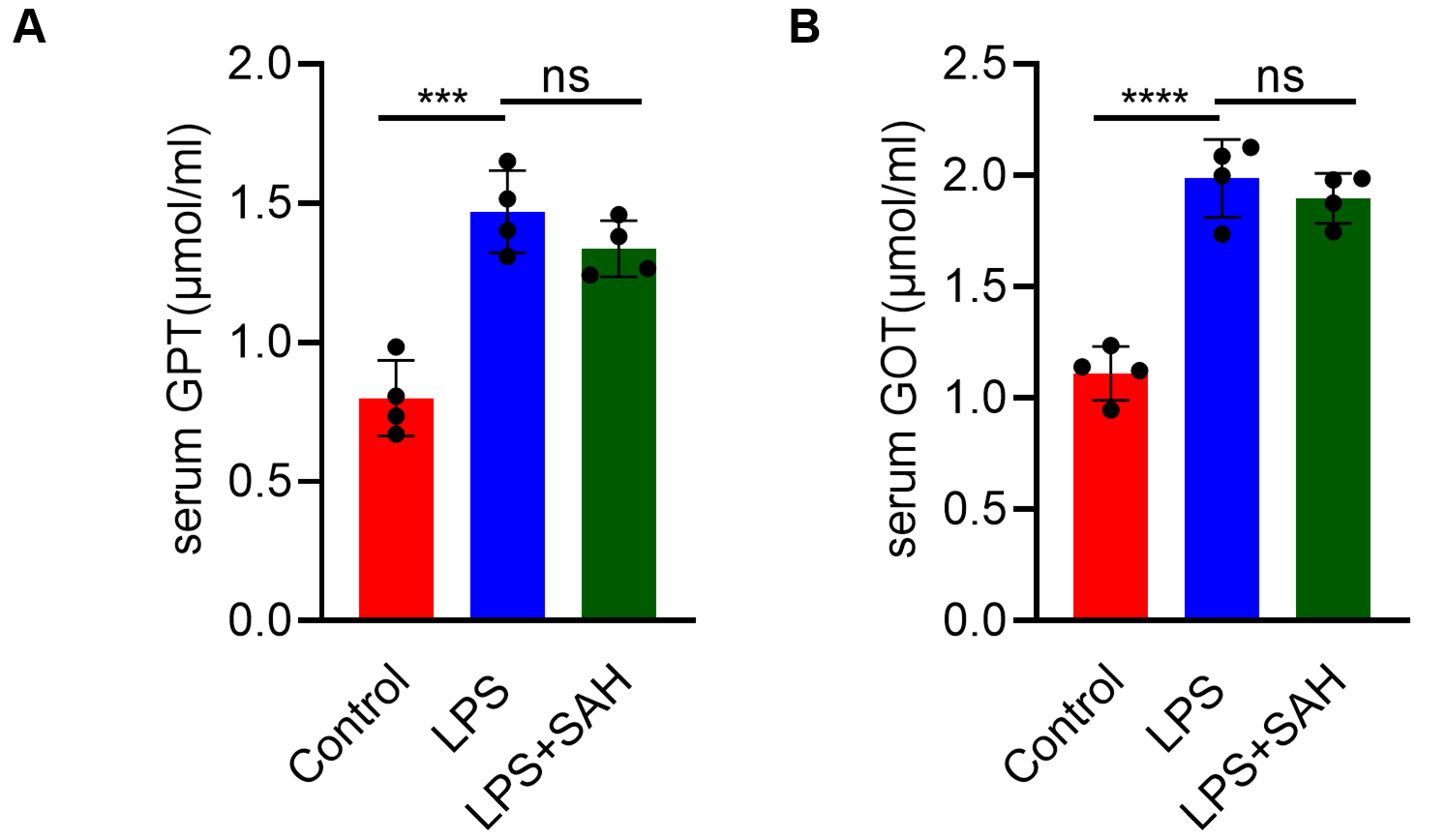

Supplement: Supplementary file 2 — Supplementary Material 2 [file 12931_2024_2894_MOESM2_ESM.tif]

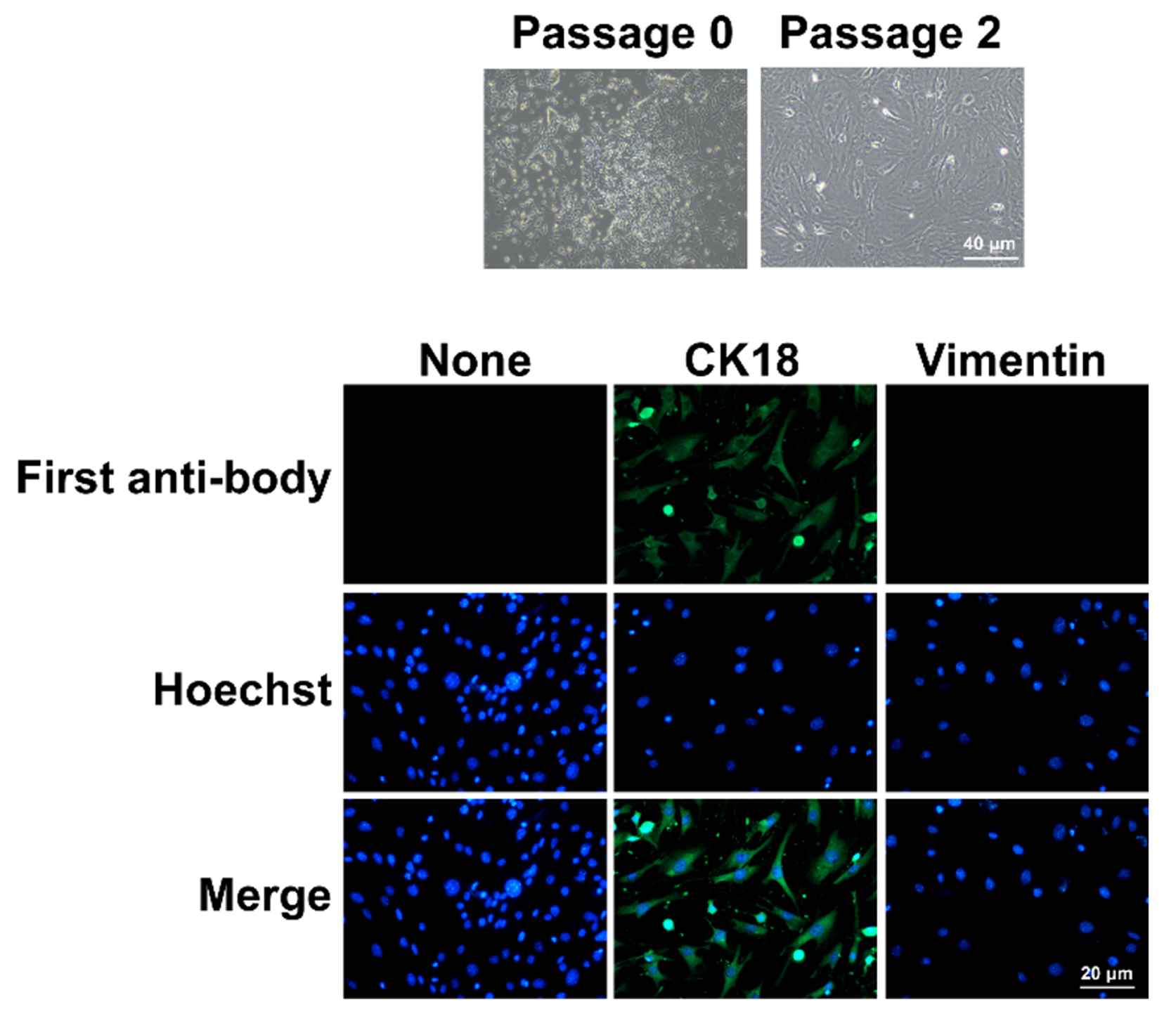

Supplement: Supplementary file 3 — Supplementary Material 3 [file 12931_2024_2894_MOESM3_ESM.tif]
